# Programmed Cell Death-Related Gene Signature Associated with Prognosis and Immune Infiltration and the Roles of HMOX1 in the Proliferation and Apoptosis were Investigated in Uveal Melanoma

**DOI:** 10.1007/s13258-024-01521-x

**Published:** 2024-05-20

**Authors:** Yubao Zhao, Liang Wang, Xiaoyan Li, Junzhi Jiang, Yan Ma, Shuxia Guo, Jinming Zhou, Yingjun Li

**Affiliations:** 1 Department of Ophthalmology, Fuyang Cancer Hospital of Fuyang Normal University, Fuyang, 236000 Anhui China; 2 School of Life Sciences, Sun Yat-Sen University, Guangzhou, 510000 Guangdong China; 3 Department of Science and Education, Fuyang Cancer Hospital of Fuyang Normal University, Fuyang, 236000 Anhui China; 4 Department of Ophthalmology, Fuyang People’s Hospital of Anhui Medical University, Fuyang, 236000 Anhui China

**Keywords:** Uveal melanoma, Programmed cell death, Prognostic signature, HMOX1, Ferrostatin, Tumor microenvironment

## Abstract

**Background:**

Uveal melanoma (UVM) is the most common primary ocular malignancy, with a wide range of symptoms and outcomes. The programmed cell death (PCD) plays an important role in tumor development, diagnosis, and prognosis. There is still no research on the relationship between PCD-related genes and UVM. A novel PCD-associated prognostic model is urgently needed to improve treatment strategies.

**Objective:**

We aim to screen PCD-related prognostic signature and investigate its proliferation ability and apoptosis in UVM cells.

**Methods:**

The clinical information and RNA-seq data of the UVM patients were collected from the TCGA cohort. All the patients were classified using consensus clustering by the selected PCD-related genes. After univariate Cox regression and PPI network analysis, the prognostic PCD-related genes were then submitted to the LASSO regression analysis to build a prognostic model. The level of immune infiltration of 8-PCD signature in high- and low-risk patients was analyzed using xCell. The prediction on chemotherapy and immunotherapy response in UVM patients was assessed by GDSC and TIDE algorithm. CCK-8, western blot and Annexin V-FITC/PI staining were used to explore the roles of HMOX1 in UVM cells.

**Results:**

A total of 8-PCD signature was constructed and the risk score of the PCD signature was negatively correlated with the overall survival, indicating strong predictive ability and independent prognostic value. The risk score was positively correlated with CD8 Tcm, CD8 Tem and Th2 cells. Immune cells in high-risk group had poorer overall survival. The drug sensitivity demonstrated that cisplatin might impact the progression of UVM and better immunotherapy responsiveness in the high-risk group. Finally, Overespression HMOX1 (OE-HMOX1) decreased the cell viability and induced apoptosis in UVM cells. Recuse experiment results showed that ferrostatin-1 (fer-1) protected MP65 cells from apoptosis and necrosis caused by OE-HMOX1.

**Conclusion:**

The PCD signature may have a significant role in the tumor microenvironment, clinicopathological characteristics, prognosis and drug sensitivity. More importantly, HMOX1 depletion greatly induced tumor cell growth and inhibited cell apoptosis and fer-1 protected UVM cells from apoptosis and necrosis induced by OE-HMOX1. This work provides a foundation for effective therapeutic strategy in tumour treatment.

**Supplementary Information:**

The online version contains supplementary material available at 10.1007/s13258-024-01521-x.

## Introduction

The uveal melanoma (UVM) is the most common intraocular malignancy of the eye in adults. UVM patients are diagnosed in approximately 7 out of one million every year (Carvajal et al. 2023; Miyamoto et al. 2012). The 5-year survival for patients with UVM is approximately 80% (Wei et al. 2022). Recently, chemotherapeutic and immunological agents have been introduced to decrease the tumor burden and increase clinical benefit (Carvajal et al. 2023; Shildkrot and Wilson. 2009). While nearly 50% patients develop metastatic disease (Sajan et al. 2023). Therefore, it is urgent to understand the biological and immunological profiles of UVM to develop novel effective therapeutic strategies.

The programmed cell death (PCD) refers to the form of cell death and can be regulated by a variety of biomacromolecules (Peng et al. 2022). Currently, the most intensively explored PCD included apoptosis, necroptosis, autophagy-dependent cell death, pyroptosis, ferroptosis, cuproptosis, alkaliptosis, lysosome-dependent cell death, netotic cell death, entotic cell death, oxeiptosis and parthanatos-dependent cell death (Liu et al. 2022a).

Apoptosis leads to cell membrane blebbing and cause cell disintegration followed by the engulfment by phagocytic housekeepers (Taylor et al. 2008). This process will not release proinflammatory cellular contents to the extracellular environment (Taylor et al. 2008). Therefore, apoptosis is regarded as non-immunogenic PCD without initiating further inflammation (Liu et al. 2022a). Inappropriate apoptosis has been regarded as a factor in many human conditions including many types of cancer (Singh and Lim. 2022). Necroptosis belongs to PCD, which involves the permeabilization of the lysosomal membrane and mitochondrial damage (Galluzzi et al. 2017). While other evidence indicated that necroptosis-deficient cancer cells are poorly immunogenic and escape therapy-triggered immunosurveillance (Galluzzi et al. 2017; Yang et al. 2016). Similarly, on the one hand, autophagy-mediated cell deaths were regarded as a form of regulated cell death depending on the autophagic machinery and functioned on suppressing the oncogenic transformation (Nassour et al. 2019). On the other hand, autophagy-mediated cell deaths played crucial role in establishing resistance to therapies in cancer, including UVM (Amaravadi et al. 2019). Autophag signature was used for evaluating the prognosis and immune infiltration as a strong predictor in UM patients (Zheng et al. 2021). Inhibiting autophagy led to increasing the cisplatin sensitivity in uveal melanoma cells (He et al. 2023). Pyroptosis is a lytic type of regulated cell death correlated with inflammation (Zhang et al. 2022b). Study have been reported that pyroptosis was involved in the pathogenesis of human hepatocellular carcinoma (Deng et al. 2022). The induction of pyroptosis inhibited the viability, migration and invasion capacity of HepG2 cells (Chu et al. 2016). Moreover, pyroptosis could accurately guide the prognosis of UVM (Cao et al. 2021). Ferroptosis is an iron-dependent and reactive oxygen species (ROS)-reliant cell death and plays an important role in the depression of tumorigenesis by removing the damaged cells (Mou et al. 2019). While study reported that increased ferroptosis induced activation and infiltration of immune cells but attenuated antitumor cytotoxic killing in glioma (Liu et al. 2022b). In UVM, ferroptosis depletion diminished hepatic metastasis in uveal melanoma (Jin et al. 2023). Cuproptosis is a form of copper-related mitochondrial cell death (Xie et al. 2023). Increased copper levels triggered tumor cell proliferation, angiogenesis, and metastasis (Oliveri. 2022). Study also have reported that the alteration of cuproptosis-related genes was related to prognosis in patients with UVM (Chen et al. 2022). More novel cuproptosis-associated biomarkers still need to be investigated. Alkaliptosis is a pH-dependent form of regulated cell death and has been recently reported as a target types of nonapoptotic cell death for cancer therapy (Liu et al. 2020). The development of lysosome-disrupting agents has been identified to induce lysosomal membrane permeabilization and release lysosome-dependent cell death (Berg et al. 2022). A recent study showed that the lysosome-dependent cell death was caused by niclosamide causes in endometrial cancer cells and tumors (Rai et al. 2023).

Zou et. al established a cell death index for prediction of survival in triple-negative breast cancer using PCD genes including netotic, entotic, oxeiptosis and parthanatos-dependent cell death. The results demonstrated that patients with high cell death index had a poor prognosis and might be sensitive to palbociclib (Zou et al. 2022). Indeed, numerous studies have revealed the association between PCD and progression of tumor. However, a comprehensive summary of the relationship between PCD and UVM remains unknown. The detailed roles of PCD in UVM have been less studied. In our study, the comprehensive bioinformatics analysis and validation experiments were performed to establish and substantiate a PCD gene signature to predict prognosis, risk stratification, and therapeutic response in UVM patients.

## Methods

### Data Sources

**Fig. 1:**
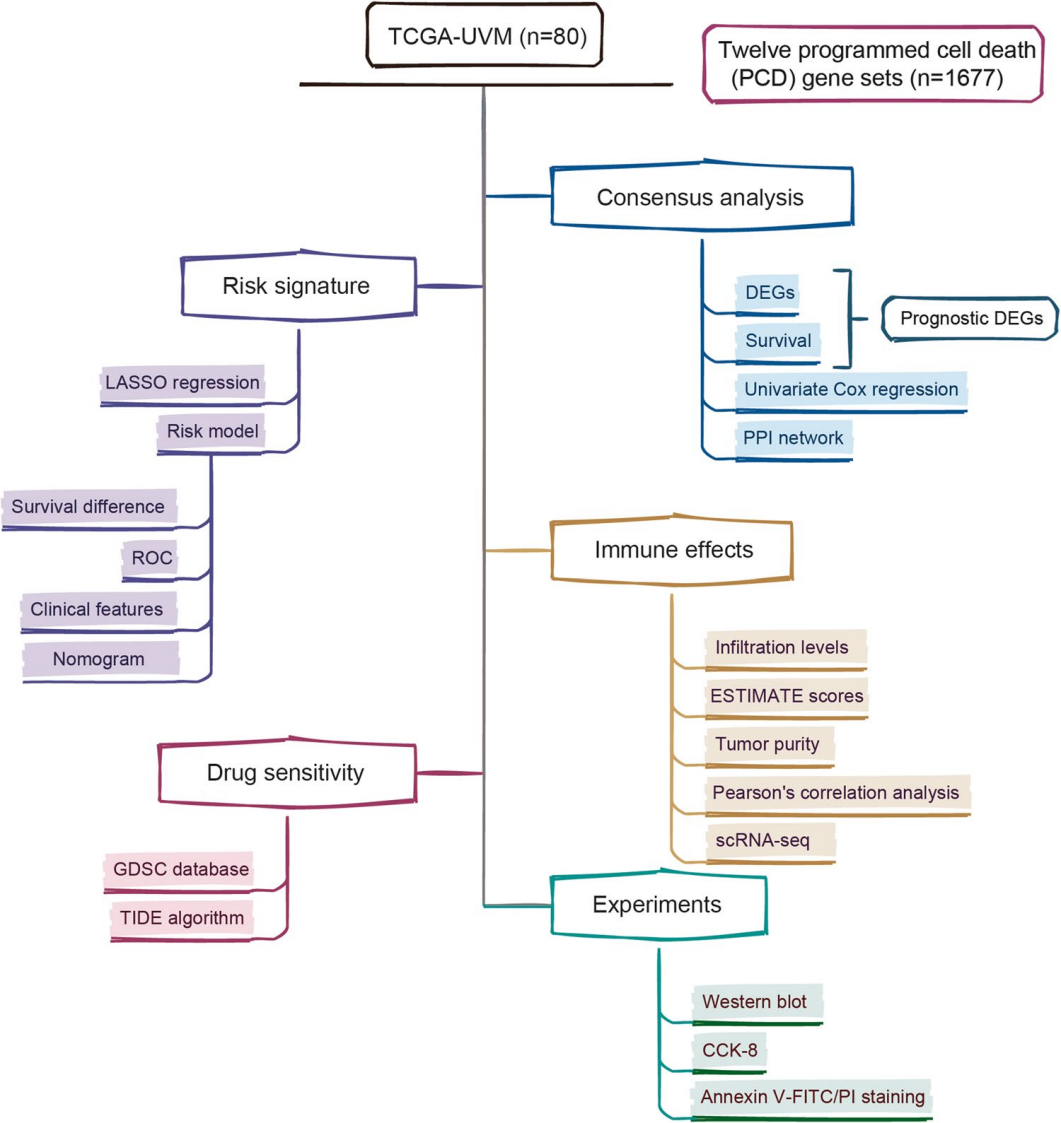
The flowchart of the present study summarizing the main design

The RNA sequencing (RNA-seq) data of UVM and adjacent normal samples were collected from the TCGA database (https://portal.gdc.cancer.gov/). This study included 80 UVM samples in the TCGA cohort. Twelve programmed cell death (PCD) gene sets were obtained from MsigDB database, HADb dataset (http://autophagy.lu/clustering/index.html), and published literature (Tang et al. 2019), including 680 apoptosis-related genes, 101 necroptosis-related genes, 45 pyroptosis genes, 86 ferroptosis-related genes, 14 cuproptosis-related genes, 8 netotic cell death-related genes, 9 parthanatos-dependent cell death-related genes, 7 alkaliptosis-related genes, 802 lysosome-dependent cell death-related genes, 222 autophagy-dependent cell death-related genes, 15 entotic cell death-related genes and 5 oxeiptosis-related genes. Ultimately, 1677 PCD genes were obtained by removing duplicate gene names. Supplementary Table 1 and Fig. 1 show more details about the source of datasets and the analysis procedure.

### Analysis of consensus cluster analysis and differentially expressed genes

All the UVM patients were divided into different groups by using “ConsensusClusterPlus” package with 1000 iterations and resampling rate of 80% (http://www.bioconductor.org/). The survival curves of different groups were generated by the Kaplan–Meier method with log-rank test using “survival” package. Differentially expressed genes (DEGs) were calculated by using “limma” package with a P value < 0.01 and log_2_(fold change) > 0.5 as significant threshold between different groups.

### Construction of the prognostic risk model

We constructed a prognostic risk score signature and model. Firstly, the univariate Cox regression analysis was conducted to extract prognostic genes, which were associated with the overall survival. Secondly, protein–protein interaction (PPI) was used to explore gene interactions and select hub genes using the search tool for the retrieval of interacting genes/proteins database (STRING, version 11.0, http://string-db.org/). Thirdly, the least absolute shrinkage and selection operator (LASSO) Cox regression analysis was used to reduce the dimension of high-latitude data through selecting the penalty parameter (λ) according to the minimum criteria. Finally, the prognostic model was constructed using the R package “glmnet” (Yu et al. 2022). The formula was as follows: risk score = Coe_CD38_ × Exp_CD38_ + Coe_TYROBP_ × Exp_TYROBP_ + Coe_CDH1_ × Exp_CDH1_ + Coe_BCL3_ × Exp_BCL3_ + Coe_HMOX1_ × Exp_HMOX1_ + Coe_CTSC_ × Exp_CTSC_ + Coe_CD5_ × Exp_CD5_ + Coe_CCR5_ × Exp_CCR5。_ And then patients were divided into high-risk and low-risk subgroups accordingly. The overall survival probability of high-risk and low-risk subgroups using the R package “survival”. Moreover, the “survivalROC” R package was employed to perform receiver operating characteristic (ROC) analysis for evaluation of the stability in the prognostic model.

### Estimation of TME cell infiltration

The composition of immune cells from the gene expression profile of complex tissues were calculated by the xCell algorithm. The infiltration level of 64 immune cells based on the expression profile data of UVM were performed using the R package “xCell” to investigate TME cell infiltration between high-risk and low-risk groups. Subsequently, the ESTIMATE algorithm was used to calculate the immune score in each patient using “estimate” package in R (Yoshihara et al. 2013).

### Drug sensitivity analysis

The genomics of drug sensitivity in cancer database (GDSC, https://www.cancerrxgene.org/) was used for drug sensitivity analysis. Eleven commonly chemotherapeutic drugs for UVM were selected from GDSC dataset according to National Comprehensive Cancer Network (NCCN) guideline (version: 1.2023), including temozolomide, Paclitaxel, Trametinib, Gemcitabine, Cisplatin, Vinblastine, Docetaxel, Docetaxel, Selumetinib, Sorafenib, Dabrafenib. The ‘oncoPredict’ package was utilized to estimate the half inhibitory concentration (IC50) values of chemotherapeutic drugs.

### The single-cell RNA-seq (scRNA-seq) analysis

The scRNA-seq data was extracted from GSE138433 database. A total of 20,153 features across 21,494 cells were obtained in 6 UVM samples after cell filtering and quality control. There were 13 subtypes with cluster-specific genes obtained with a resolution of 0.3 using “seurat” package (version 4.0.3) in R software. The CellMarker (http://xteam.xbio.top/CellMarker/download.jsp) and panglaodb (https://panglaodb.se/markers.html?cell_type=%27all_cells%27) database were used to annotate these subtypes, including "Endothelial cells", "Fibroblasts", "Natural killer T cells", "Regulatory T cells", "Monocytes", "Melanoma cancer cells", "Mast cells", "Astrocytes", "Neural stem/precursor cells", "Macrophages", "B cells", "Plasmacytoid dendritic cells" and "Th1 cells".

### Cell lines and cell culture

The UVM cell lines (MM68, MP65) were provided by Stem Cell Bank, Chinese Academy of Sciences and were cultured in RPMI-1640 medium, supplemented with 20% fetal bovine serum (Welgene) and 1% penicillin/streptomycin (Welgene).

### Cell transfection

The short hairpin RNAs (shRNAs) used to silence HMOX1 (sh-HMOX1) in UVM cells and its negative control vector (sh-NC). And the HMOX1 overexpressing vector (OE- HMOX1) and OE-NC were synthesized by GenePharma (Shanghai, China). sh-HMOX1 and sh-NC vectors were induced into cells with 7 µg/mL polybrene. The OE-HMOX1and OE-NC were transfected into cells using lipofectamine™ 2000 transfection reagent (Invitrogen). Additionally, ZnPP, Z-Vad-fmk, necrostatin-1, ferrostatin-1 (fer-1), deferoxamine (DFO) and chloroquine were obtained from Sigma-Aldrich and dissolved in DMSO. Tumor cells were incubated with final concentrations of 10 µmol/L ZnPP, 50 µmol/L Z-Vad-fmk, 50 µmol/L necrostatin-1, 1 µmol/L fer-1, 50 µmol/L DFO, 50 µmol/L chloroquine for 1 h (Wang et al. 2023).

### Cell proliferation assay

The cells were seeded in flat-bottomed 96-well plates with a density of 1,000 cells per well. Then 10 µl of Cell Counting Kit-8 (CCK-8; Dojindo, CK04) was added to each well for 4 h. We determined the optical density at 450 nm using a microplate reader (Thermo Scientific).

### Western blot

Cells were homogenised with lysis buffer with protease inhibitor cocktail (Sigma Aldrich). Then the protein concentration was determined with the bicinchoninic acid protein assay kit (Sigma-Aldrich, #BCA1-1KT). The samples were separated by 10% SDS-PAGE and transferred onto nitrocellulose membranes (Millipore). The membranes were blocked with 5% BSA for 2 h at room temperature and then incubated with primary antibodies targeting HMOX1, BCL2, BAX or GAPDH (No. #5174, Cell Signaling Technology) overnight at 4 °C. After washing by PBST, the membranes were incubated with a secondary antibody conjugated to a fluorescent tag (Sigma Aldrich, #A0545-1ML, dilution ratio 1:200). The band signals were visualized and quantified using the Odyssey Infrared Imagining System (LI-COR, USA). The specific protein bands were calculated using the Image J software with the density values.

### Annexin V-FITC/Propidium iodide staining

The Annexin V-FITC/propidium iodide (PI) assay kit with a flow cytometry (FACSCanto II; BD Bioscience) was used to measure the normal, apoptotic, and necrotic of MP65 cells according to the manufacturer’s instructions.

### Statistical analysis

All the data were presented as means ± standard deviation (SD). Student’s two-tailed *t* test was used to determine significant differences in two groups and one-way ANOVA followed with Tukey’s test was used for test in multiple groups. *P* < 0.05 was considered statistically significant.

## Results

### Identification of the novel prognostic genes in UVM

**Fig. 2:**
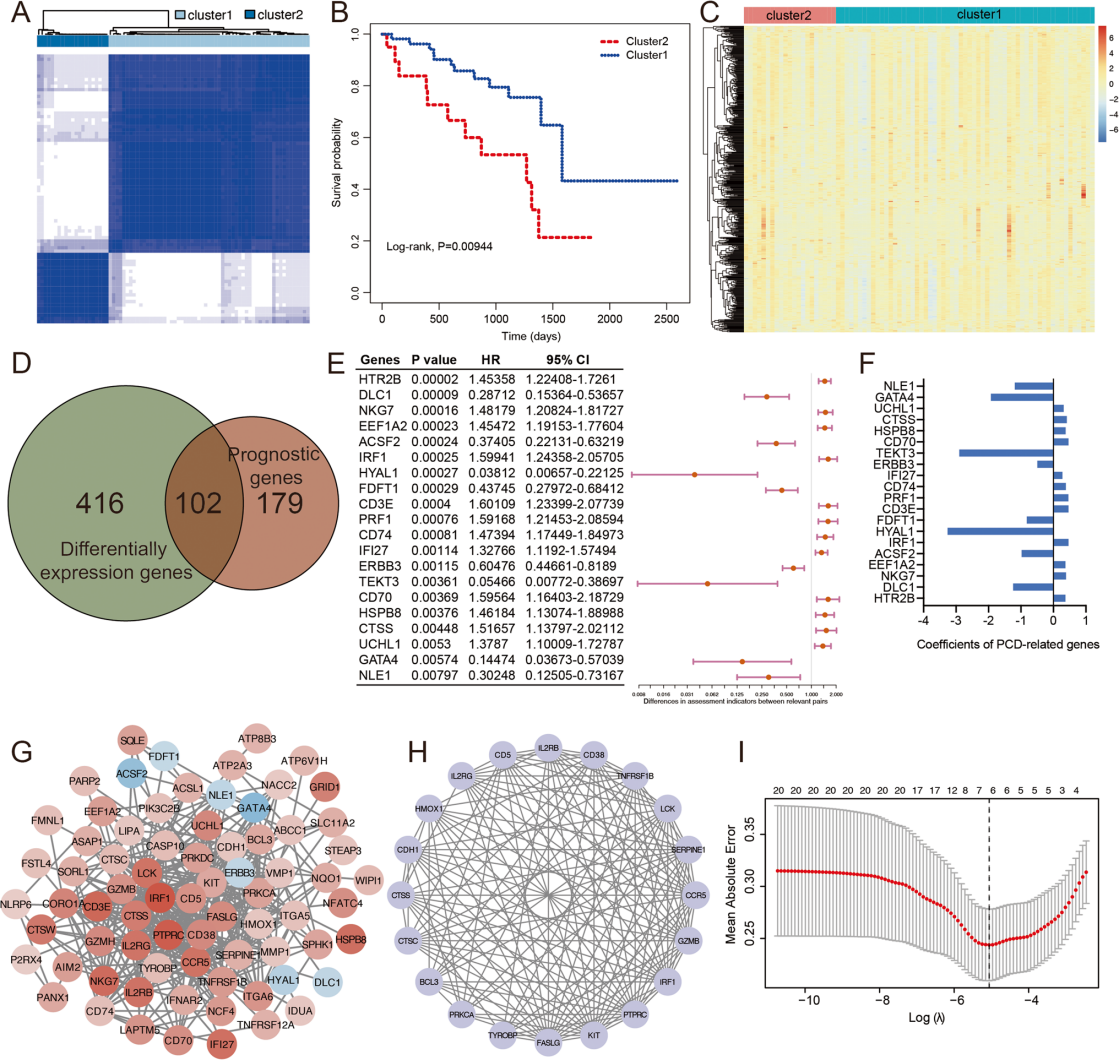
Screening of the PCD-related genes in the UVM patients from the TCGA database. (**A**) The heatmap of consensus matrix defining two clusters (k = 2). (**B**) The Kaplan–Meier survival curve for overall survival between cluster1 and cluster2. (**C**) Differences in expression levels of PCD-related genes between the two distinct clusters. (**D**) Venn diagram showing the intersection of genes between differentially expression genes and prognostic genes. (**E**) The univariate Cox regression analysis indicating PCD-related genes correlated with the overall survival. (**F**) The coefficients of PCD-related genes. (**G**) Protein–protein interaction analysis of PCD-related genes. (**H**) Hub genes in protein–protein interaction network. (**I**) LASSO regression model based on the hub genes

The overview of various steps involved in this work are outlined in Fig. 1. To identify novel prognostic genes, the consensus cluster analysis was used to classify molecular subtypes with distinct prognosis. UVM patients were divided into two distinct molecular subtypes based on overall gene expression (Fig. 2A). Cluster 2 had poorer prognosis than cluster 1 (*P* = 0.00944, Fig. 2B). In order to look at the transcription between two molecular subtypes. The results showed that the expression differences of 618 genes were statistically significant between cluster 1 and cluster 2, with *P* < 0.01 and | log_2_(fold changes) | > 0.5 as the threshold (Fig. 2C). Furthermore, we performed a univariate Cox regression analysis to assess the prognostic value. A total of common 102 DEGs were detected as novel DEGs through the intersection of DEGs of two molecular subtypes and genes associated with overall survival (Fig. 2D-E). The coefficients of PCD-related genes were shown in Fig. 2F. Furthermore, the protein–protein interaction (PPI) network was constructed with STRING database for interaction relationships of novel DEGs (Fig. 2G). The larger the degrees, the greater importance of the corresponding genes in the network found. The top 20 corresponding genes were considered as hub genes and visualized in Fig. 2H. Subsequently, the LASSO algorithm was used to obtain the coefficient of novel DEGs (Fig. 2I). The minimum standard, 8 genes (CD38, TYROBP, CDH1, BCL3, HMOX1, CTSC, CD5 and CCR5) were selected to construct a prognostic risk score signature. And the risk score of each UVM patient was calculated as follows: risk score = 0.18246 × Exp_CDH1_—0.11415 × Exp_TYROBP_—0.58195 × Exp_CD38_ + 0.20079 × Exp_BCL3_ + 0.23549 × Exp_HMOX1_ + 0.42475 × Exp_CTSC_ + 0.03411 × Exp_CD5_ + 0.09686 × Exp_CCR5._

### Risk scores is an independent prognostic factor

**Fig. 3:**
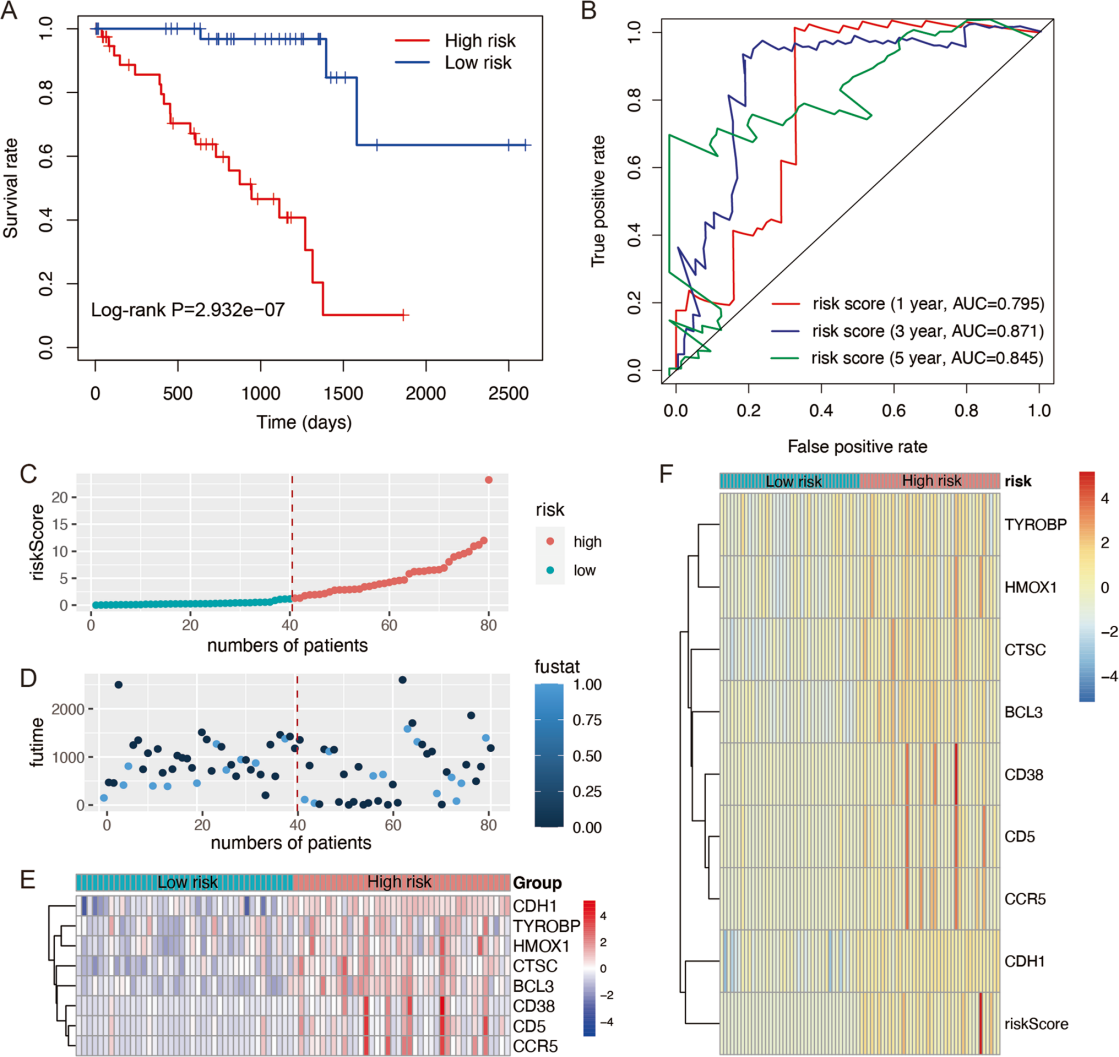
Construction of the PCD genes-based independent prognostic risk model. (**A**) The Kaplan–Meier curve of the overall survival in the high-risk and low-risk groups. (**B**) The ROC curves to evaluate the predictive efficiency at 1, 3 and 5 year. (**C**-**E**) Distribution of the risk scores, survival status and expression levels of 8 signature genes. (**F**) The risk scores and RNA expression between high- and low-risk groups

All UVM patients were stratified into high-risk (*n* = 40) and low-risk (*n* = 40) groups, according to the median value (1.223956) of the risk score. To verify the prognostic value of risk grouping in UVM patients, the Kaplan–Meier survival analysis was conducted to evaluate association between overall survival and risk scores. The results demonstrated that the overall survival of patients in the high-risk group was significantly lower than that in the low-risk group (*P* = 2.932e-07, Fig. 3A). Furthermore, the ROC curves were used to evaluate the specificity and sensitivity of the prognostic signatures in risk model. The areas under the ROC curve (AUC) for 1-, 3- and 5-year overall survival were 0.795, 0.871 and 0.845, respectively in TCGA cohort (Fig. 3B). The distribution of risk score, survival status, and the expression levels of prognostic signature between high- and low-risk groups were shown in Fig. 3C-F.

### Integrated prognostic model based on PCD prognostic risk signature and clinicopathological features

**Fig. 4:**
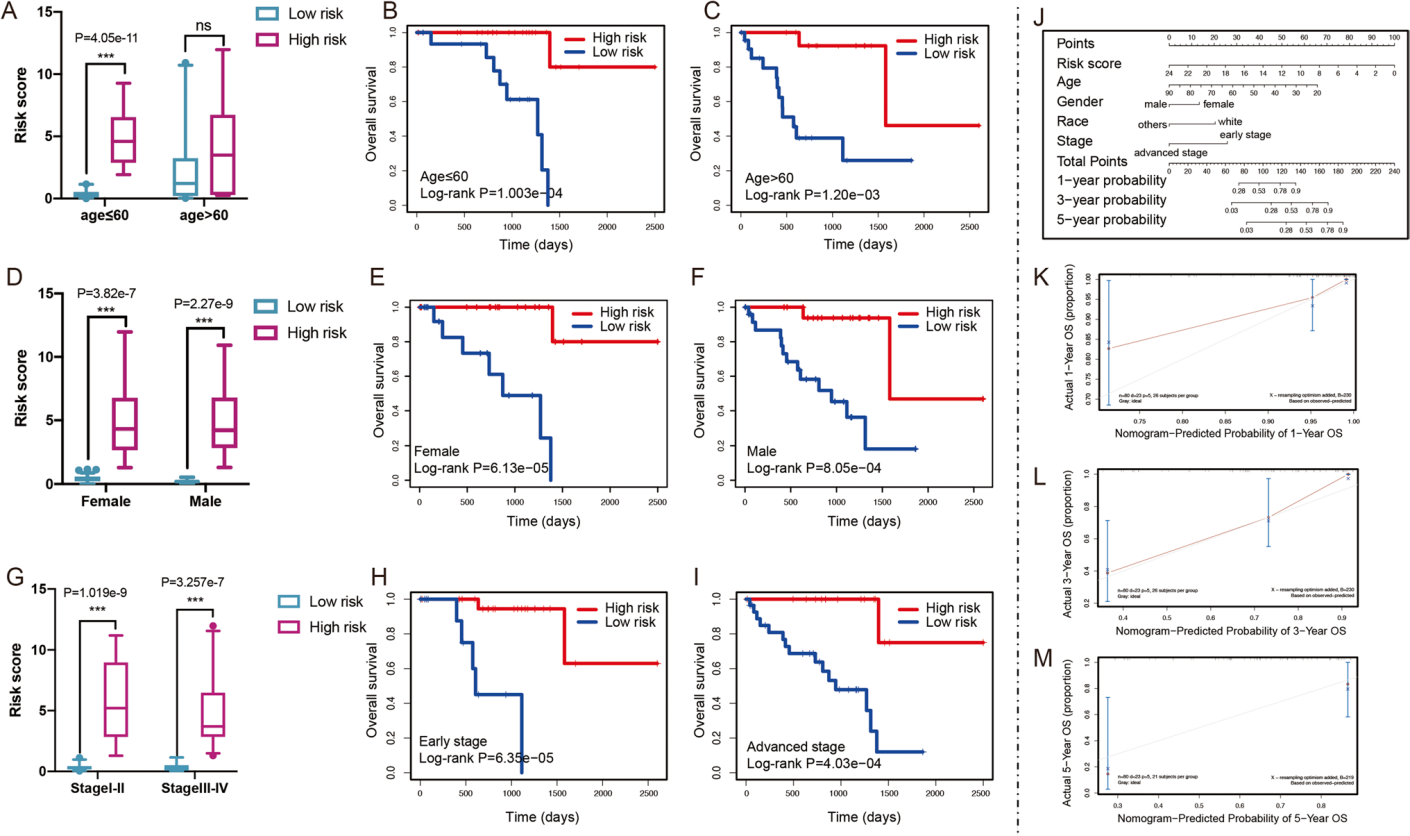
Clinicopathologic characteristics in UVM. (**A**) Difference of risk scores in age features. (**B**) The Kaplan–Meier curve indicating the prognosis value in younger patients (age ≤ 60). (**C**) The Kaplan–Meier curve indicating the prognosis value in older patients (age > 60). (**D**) Difference of risk scores in gender groups. (**E**) The Kaplan–Meier curve of overall survival between the two risk groups in female patients. (**F**) The Kaplan–Meier curve of overall survival between the two risk groups in male patients. (**G**) Difference of risk scores in stage characteristic. (**H**) Survival analysis for patients with high- and low-risk group using Kaplan–Meier curves in early stage. (**I**) Survival analysis for patients with high- and low-risk group using Kaplan–Meier curves in advanced stage. (**J**) Nomogram for predicting the 1-, 3-, and 5-year overall survival of patients with UVM. (K-M) Calibration curves of the nomogram for predicting of 1-, 3-, and 5-year overall survival

To explore the association of risk score with clinical characteristics, the differences of risk scores were calculated between high- and low-risk group in clinical variables, including age, gender and stage. The results showed that patients with age ≤ 60 had higher risk scores in high-risk group (Fig. 4A, *P* = 4.05e-11). A higher risk score was associated with significantly better prognosis both patients with age ≤ 60 (Fig. 4B, *P* = 1.003e-4) and age > 60 (Fig. 4C, *P* = 1.20e-3). Then, dramatically significant differences were found in females (Fig. 4D, *P* = 3.82e-7) and males (Fig. 4D, *P* = 2.27e-9). The survival of patients in the low-risk group was significantly longer than those in the high-risk group in terms of both females (Fig. 4E, *P* = 6.13e-5) and males (Fig. 4F, *P* = 8.05e-4). In addition, patients with early stage (stage I and II) had higher risk scores in high-risk group compared with low-risk group (Fig. 4G, *P* = 1.019e-9). Patients with advanced stage (stage III and IV) also had higher risk scores in high-risk group compared with low-risk group (Fig. 4G, *P* = 3.257e-7). Compared with the low-risk group, patients in the high-risk group had better prognosis both early-stage UVM (Fig. 4H, *P* = 6.35e-5) and advanced-stage tumors (Fig. 4I, *P* = 4.03e-4).

**Fig. 5:**
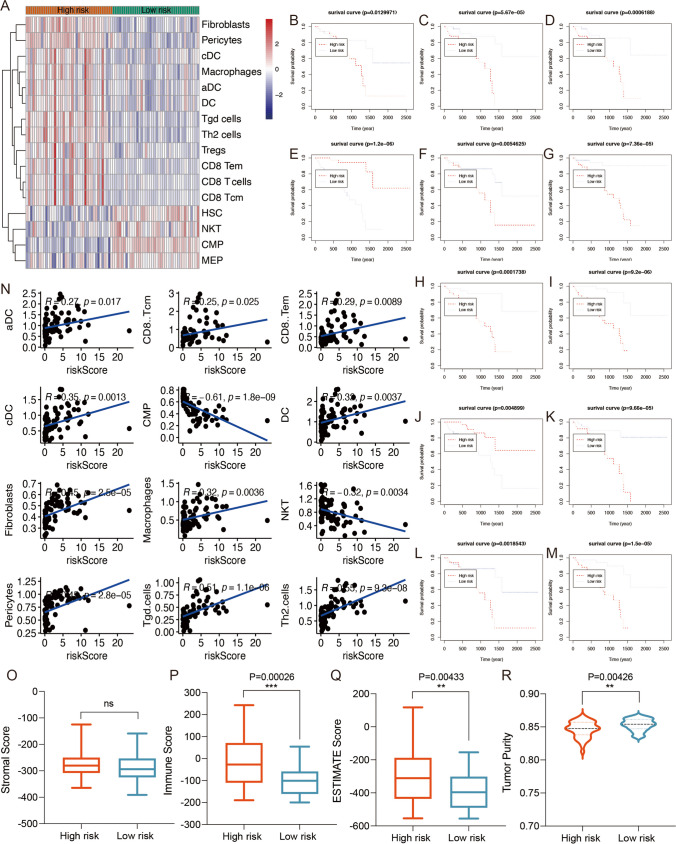
Immune infiltration analysis among different risk groups. (**A**) The relative abundance of immune cells based on xCell in the different risk groups. The Kaplan–Meier survival plotter of aDC (**B**), DC (**C**), cDC (**D**), CMP (**E**), pericytes (**F**), fibroblasts (**G**), CD8 Tcm (**H**), Tgd cells (**I**), NKT (**J**), CD8 Tem (**K**), Macrophages (**L**), Th2 cells (**M**). (**N**) Correlation between the risk scores and tumor immune cells in UVM. The correlation of risk scores and stromal score (**O**), immune score (**P**), ESTIMATE score (**Q**) and tumor purity (**R**)

To establish a quantitative approach for PFS prediction, we built a nomogram through combining risk score and other clinicopathological features (Fig. 4J). The calibration plot was used to evaluate the reliability of the nomogram. As a result, the line-segments were closed to the 45° line at 1-year (Fig. 4K), 3-year (Fig. 4L) and 5-year overall survival (Fig. 4M), suggesting that the observation in our cohort were excellent agreement with the prediction.

### Immune infiltration analysis based on the prognostic risk signature

Subsequently, we evaluated the impact of prognostic risk signature on immune microenvironment in UVM, the difference of tumor immune infiltration between high-risk and low-risk group were calculated to estimate the proportions of 64 distinct immune cell phenotypes by using “xCell” algorithm.

Our results revealed that a total of 16 immune cell phenotypes were statistically different between high-risk and low-risk group (Fig. 5A). To investigate the association with overall survival and the abundance of immune cell phenotypes, the survival curves were performed. Compared with low-risk group, immune cells in high-risk group had poorer overall survival, including aDC (*P* = 0.012997, Fig. 5B), DC (*P* = 0.0054625, Fig. 5C), cDC (*P* = 9.66e-05, Fig. 5D), pericytes (*P* = 0.0001738, Fig. 5F), fibroblasts (*P* = 7.36e-05, Fig. 5G), CD8 Tcm (*P* = 5.67e-05, Fig. 5H), Tgd cells (*P* = 9.2e-06, Fig. 5I), CD8 Tem (*P* = 0.0006188, Fig. 5K), macrophages (*P* = 0.0018543, Fig. 5L), Th2 cells (*P* = 1.5e-05, Fig. 5M). In contract, CMP (*P* = 1.2e-06, Fig. 5E) and NKT (*P* = 0.004899, Fig. 5J) cells in high-risk group showed better overall survival compared with low-risk group. We further investigated the correlation between the risk scores and infiltrated immune cell phenotypes. Our results showed that the risk score was positively correlated with aDC (r = 0.27, *P* = 0.017), DC (r = 0.32, *P* = 0.0037), cDC (r = 0.35, *P* = 0.0013), pericytes (r = 0.45, *P* = 2.8e-05), fibroblasts (r = 0.45, *P* = 2.5e-05), CD8 Tcm (r = 0.25, *P* = 0.025), Tgd cells (r = 0.51, *P* = 1.1e-06), CD8 Tem (r = 0.29, *P* = 0.0089), macrophages (r = 0.32, *P* = 0.0036), Th2 cells (r = 0.55, *P* = 9.3e-08), as shown in Fig. 5N. In addition, a negative correlation was observed between the risk score and CMP (r = -0.61, *P* = 1.8e-09) and NKT (r = -0.32, P = 0.0034). Furthermore, we next explored the relationship between the genes in risk model and infiltrated immune cell phenotypes. Our immune infiltration results showed that CTSC was significantly correlated with all the immune cell phenotypes and it was the most positively correlated with Tgd cells (r = 0.77, *P* = 2.5e-16, Supplementary Table 4). Then we found that HMOX1 was the most positively correlated with macrophages (r = 0.753, *P* = 3.7e-15) and followed by aDC (r = 0.748, *P* = 7.3e-15, Supplementary Table 4).

Then, we evaluated the TME scores of high-risk and low-risk group using ESTIMATE algorithm. The results demonstrated no significant difference in stromal score (Fig. 5O). However, the patients in the high-risk group had a higher immune score (*P* = 0.00026, Fig. 5P), ESTIMATE score (*P* = 0.00433, Fig. 5Q) and tumor purity (*P* = 0.00426, Fig. 5R). These results revealed a potential correlation between the infiltration of immune cell phenotypes and risk score. Next, scRNA analysis was used to investigate the relationship between immune cells and genes in risk model.

### ScRNA-seq analysis

For a further exploration of the function of genes in risk model, scRNA analysis were performed by using GSE138433 database. A total of 20,153 features across 21,494 cells were obtained in 6 UVM samples after cell filtering and quality control. There were 13 subtypes with cluster-specific genes obtained with a resolution of 0.3 using “seurat” R package. Then the cells were assigned into 13 major cell subtypes, including "Endothelial cells", "Fibroblasts", "Natural killer T cells", "Regulatory T cells", "Monocytes", "Melanoma cancer cells", "Mast cells", "Astrocytes", "Neural stem/precursor cells", "Macrophages", "B cells", "Plasmacytoid dendritic cells" and "Th1 cells" (Supplementary Fig. 3A). In scRNA-seq results, we found that the accumulation of CTSC and CDH1 expression positive rate were found in multiple cells. And the HMOX1 expression positive rate on macrophages and Th1 cells were higher than others (Supplementary Fig. 3B). Interestingly, the immune infiltration results showed HMOX1 was positively correlated with macrophages (r = 0.753, *P* = 3.7e-15, Supplementary Table 4), suggesting that HMOX1 might play important role in macrophages in UVM. After that, we next screened potential correlation between risk score and therapy response of antineoplastic drugs from GDSC dataset and TIDE algorithm.

### Evaluation of immunotherapy and chemotherapy response

**Fig. 6:**
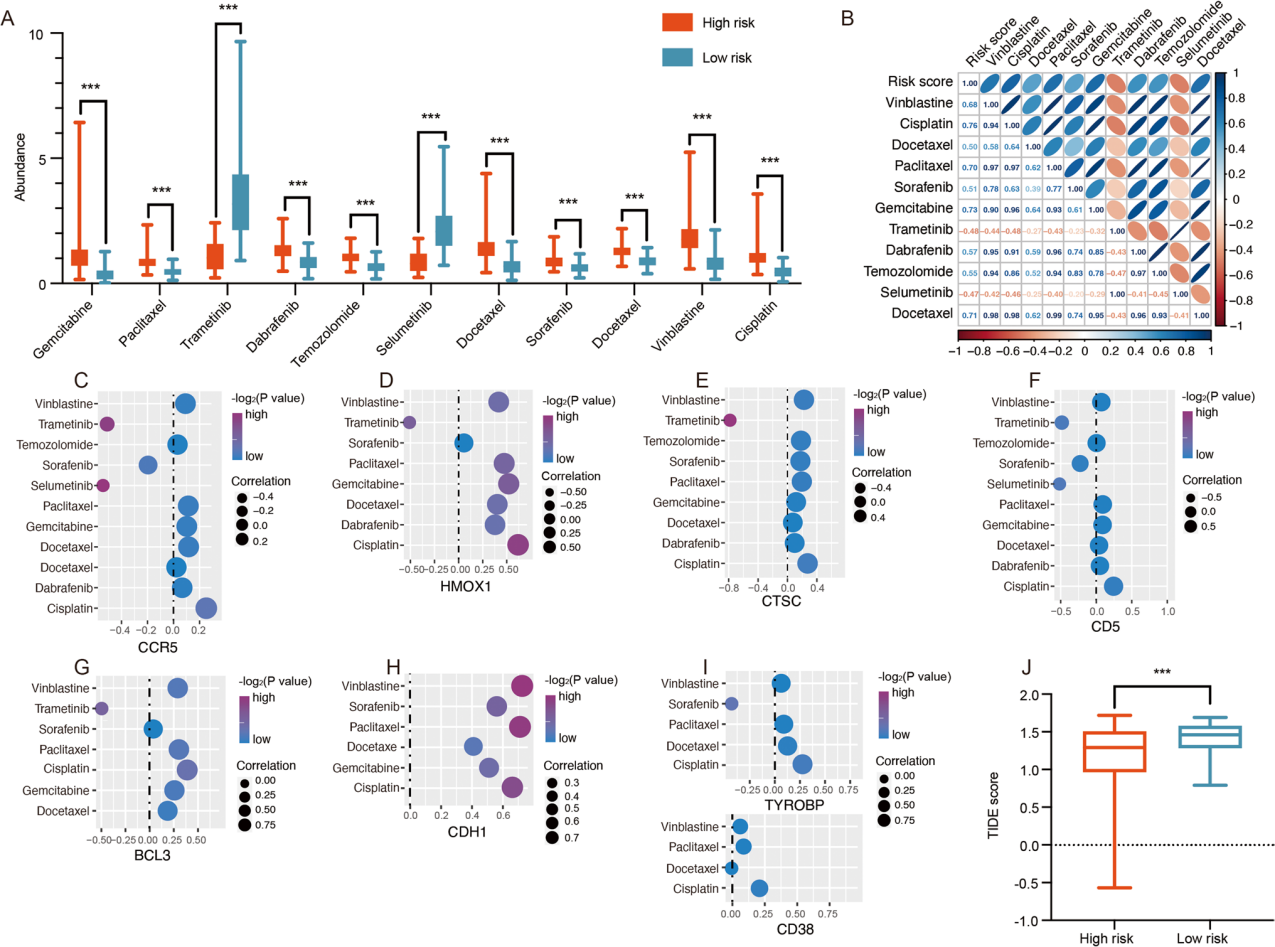
Drug sensitivity analysis of high- and low-risk groups. (**A**) Anticancer drug response differences in the different risk groups. (**B**) The relationship of risk scores and chemotherapeutic drugs. The correlation between chemotherapeutic drugs and the expression levels of CCR5 (**C**), HMOX1 (**D**), CTSC (**E**), CD5 (**F**), BCL3 (**G**), CDH1 (**H**), TYROBP (**I**), CD38 (**I**). (**J**) Difference in TIDE scores between high- and low-risk groups

To explore the effect of prognostic risk signature on chemo-drug or immune checkpoint inhibitors sensitivity, the GDSC database and TIDE algorithm were used to analyze the differences of common antineoplastic drugs between high- and low-risk group. The results revealed that 11 chemotherapeutic drugs assessed significantly differed between the two groups. The patients had lower IC50 value in the low-risk group and were more sensitive to 9 chemotherapeutic drugs, including vinblastine, cisplatin, docetaxel, paclitaxel, sorafenib, gemcitabine, dabrafenib, temozolomide and docetaxel (Fig. 6A). While trametinib and selumetinib drugs showed lower IC50 value in the high-risk group (Fig. 6A). Furthermore, the correlation of the risk scores and IC50 value of chemotherapeutic drugs were performed by Pearson's correlation analysis. The results indicated a significant positive correlation between risk scores and chemotherapeutic drugs, including vinblastine (r = 0.6762, *P* = 1.209e-11), cisplatin (r = 0.7571, *P* = 1.132e-15), docetaxel (r = 0.5009, *P* = 3.251e-6), paclitaxel (r = 0.7017, *P* = 8.852e-13), sorafenib (r = 0.5087, *P* = 2.173e-6), gemcitabine (r = 0.7291, *P* = 3.959e-14), dabrafenib (r = 0.5699, *P* = 5.539e-8), temozolomide (r = 0.5520, *P* = 1.741e-7), docetaxel (r = 0.7073, *P* = 4.903e-13). The significant negative correlation of risk scores with trametinib (r = -0.4774, *P* = 1.059e-5) and selumetinib (r = -0.4701, *P* = 1.461e-13) were found (Fig. 6B), suggesting that higher risk scores may be associated with more sensitive to drugs. In addition, we predicted the sensitivity of common anti-cancer drugs on prognostic signature, including CCR5 (Fig. 6C), HMOX1 (Fig. 6D), CTSC (Fig. 6E), CD5 (Fig. 6F), BCL3 (Fig. 6G), CDH1 (Fig. 6H), TYROBP and CD38 (Fig. 6I). Among the 11 chemotherapeutic drugs associated with risk score, a total of 5 genes were correlated with over half of the chemotherapy drugs, including CCR5, CD5, CTSC, HMOX1 and BCL3.

To predict possible tumor treatment responses of immune checkpoint inhibitors, the sensitivity of patients to immunotherapy were evaluated between high- and low-risk group by using TIDE algorithm. Our results showed that the patients in high-risk group had lower TIDE scores than that in low-risk group (Fig. 6J, *P* = 0.002447), suggesting the better immunotherapy responsiveness in the high-risk group. The correlation demonstrated better relationship between TIDE scores and biomarkers with higher correlation coefficient values, including CD5, CCR5, CD38, TYROBP and HMOX1 (Supplementary Table 2).

Receiver operating characteristic (ROC) analysis was used to evaluate the sensitivity and specificity of risk scores in predicting prognosis. The results identified CDH1 (AUC = 0.9230), HMOX1 (AUC = 0.9024) and BCL3 (AUC = 0.867) with higher accuracy of prognosis (Supplementary Table 3). Based on these observations, we found that HMOX1 might be more sensitivity respond to chemotherapy and immunotherapy and represented high sensitivity and specificity for prognostic prediction in risk model. We next focused on the biological function of HMOX1 in UVM.

### Effects of HMOX1 on the proliferation and apoptosis of UVM cells

**Fig. 7:**
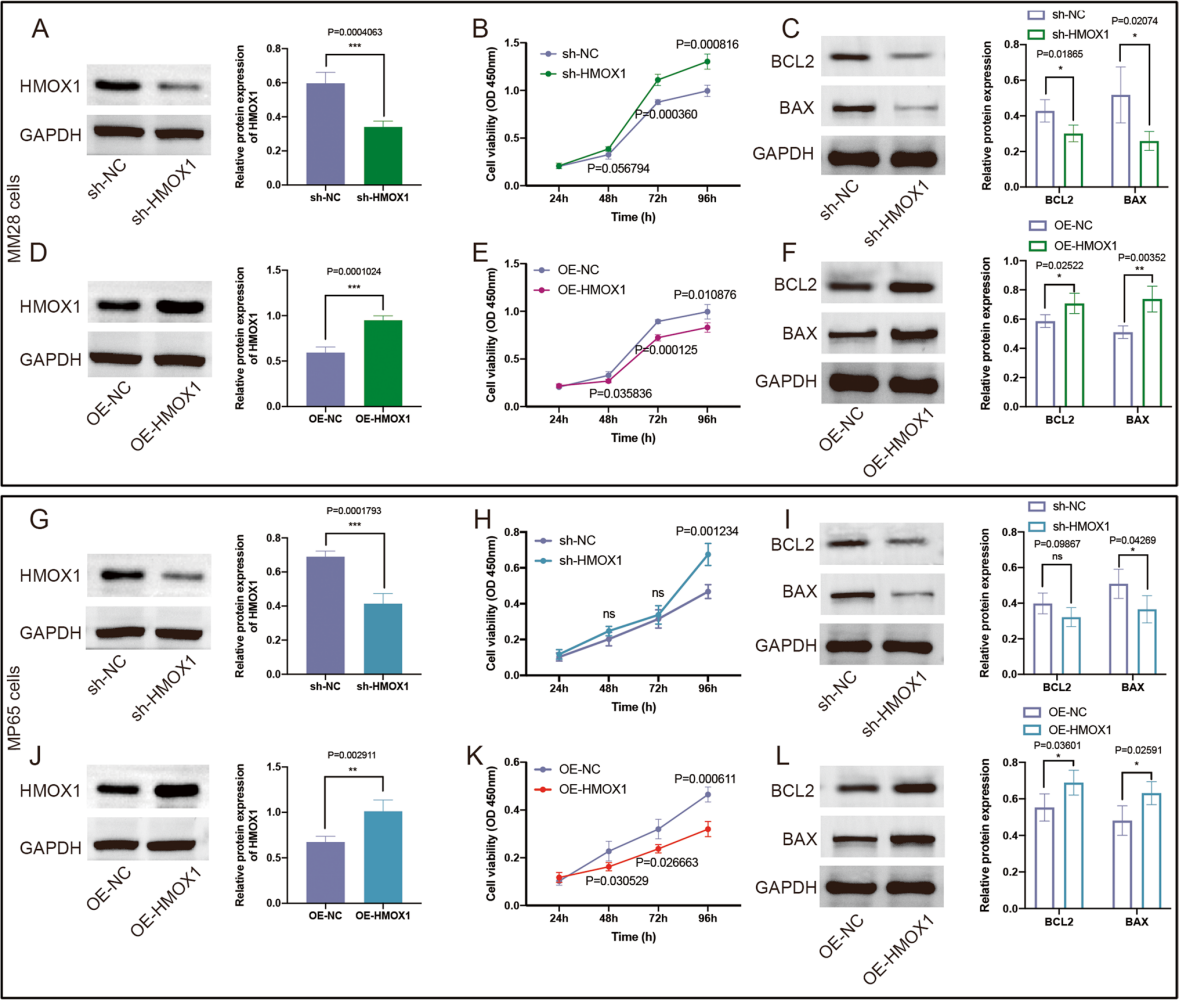
Effect of HMOX1 on the proliferation and apoptosis in UVM cells. (**A**) The western blotting was used to confirm the knockdown efficiency of HMOX1 in MM28 cells (*n* = 3). (**B**) CCK-8 assays displaying cell growth after infection with sh-HMOX1 or sh-NC in MM28 cells (*n* = 3). (**C**) Effects of HMOX1 knockout on the expression of BCL-2 and BAX in MM28 cells (*n* = 3). (**D**) The western blotting was used to confirm the overexpression efficiency of HMOX1 in MM28 cells (*n* = 3). (**E**) CCK-8 assays indicating cell growth after infection with OE-HMOX1 or OE-NC in MM28 cells (*n* = 3). (**F**) Effects of HMOX1 overexpression on the expression of BCL-2 and BAX in MM28 cells (*n* = 3). (**G**) HMOX1 levels in MP65 cells were evaluated by western blot analysis after transduction with sh-HMOX1 or sh-NC (*n* = 3). (**H**) HMOX1 silencing increased the growth of MP65 cells (*n* = 3). (**I**) HMOX1 silencing inhibited the expression of pro-apoptotic proteins BCL2 and BAX in MP65 cells (*n* = 3). (**J**) HMOX1 levels in MP65 cells were evaluated by western blot analysis after transduction with OE-HMOX1 or OE-NC (*n* = 3). (**K**) Overexpression of HMOX1 decreased the growth of MP65 cells (*n* = 3). (**L**) Overexpression of HMOX1 induced the expression of pro-apoptotic proteins BCL2 and BAX in MP65 cells (*n* = 3)

To explore the impact of HMOX1 on cell proliferation, UVM cells expressing sh-HMOX1 or sh-NC were seeded into 96-well plates and CCK-8 assay was used to measure cell proliferation. The results of western blot showed that the expression of HMOX1 was significantly reduced when sh-HMOX1 were transfected into MM28 cells (Fig. 7A) and MP65 cells (Fig. 7G). HMOX1 silencing significantly promoted MM28 cell growth (Fig. 7B) and inhibited cell apoptosis (Fig. 7C). The cell survival of MP65 cells was significantly increased in HMOX1-silenced groups as shown in Fig. 7H. Knockdown of HMOX1 inhibited MP65 cell apoptosis (Fig. 7I). Furthermore, HMOX1 expression was increased when UVM cells were transfected with OE-HMOX1, including MM28 (Fig. 7D) and MP65 cells (Fig. 7J). The overexpression of HMOX1 inhibited significant promotions in cell proliferation (Fig. 7E) and induced cell apoptosis (Fig. 7F) in MM28. The similar results were shown in MP65 cells (Fig. 7K-L).

To investigate whether HMOX1 contributed to cell death, ZnPP (an HMOX1-specific inhibitor, 10 mmol/L) was used due to its inhibitory activity on HMOX1. Compared with the negative control, the cell viability in MP65 cells transfected into sh-HMOX1 were significantly increased (*P* = 0.00123). The results also showed that pretreatment with Znpp significantly increased the cell viability (*P* = 0.000587, Supplementary Fig. 1A). Furthermore, overexpression of HMOX1 contributed to the less cell viability (*P* = 0.000611), and the treatment of ZnPP effectively prevented decreased cell viability induced by OE-HMOX1 in MP65 cells (*P* = 0.002721, Supplementary Fig. 1B).

In order to assess the involvement of ferroptosis in cell death induced by OE-HMOX1, Annexin V-FITC/PI staining was used to distinguish into normal, apoptotic, and necrotic cells of MP65 cells using an Annexin V-FITC/PI assay kit with a flow cytometry. Our results showed that fer-1 (*P* = 0.000458) and DFO (*P* = 0.000534) both prevented decreased cell viability induced by OE-HMOX1 (Supplementary Fig. 2A-B). Additionally, flow cytometry results showed that fer-1 completely protected MP65 cells from apoptosis caused by OE-HMOX1. Fer-1 and DFO also protected MP65 cells from OE-HMOX1-induced necrosis (Supplementary Fig. 2C-E). In addition, multiple PCD inhibitors were selected to investigate which type of cell death was predominant, including Z-Vad-fmk, necrostatin-1, fer-1, and chloroquine. The results of rescue experiment demonstrated that the increased cell apoptosis of MP65 cells with OE-HMOX1 were inhibited by necrostatin (*P* = 0.01832) and fer-1 (*P* = 6.0693E-04, Supplementary Fig. 2F-G). The OE-HMOX1-induced necrosis of MP65 cells were also suppressed by fer-1 (*P* = 0.004519, Supplementary Fig. 2F, H).

## Discussion

UVM is a highly malignant tumor. The currently the main treatments for UVM are surgery, radiation therapy, laser therapy, chemotherapy and immunotherapy (Chattopadhyay et al. 2016). While the survival rates for this cancer are far from satisfactory, due to the high rate of recurrence and metastasis. At the same time, programmed cell death, including multiple forms of cell death, had been proven to promote or inhibit progression of cancer in many tumor types (Dai et al. 2021; Kari et al. 2022). For instance, Tang et. al prognostic and diagnostic models based on ferroptosis and iron-metabolism genes in hepatocellular carcinoma (Tang et al. 2020). In glioma, cuproptosis-related signature might be used for the prediction of the prognosis, biological features, and appropriate treatment (Wang et al. 2022a). Another study showed that pyroptosis-related signature was a valuable signature in the identification of populations sensitive to immune checkpoint inhibitors in muscle-invasive bladder cancer (Zhang et al. 2022a). Study have showed that programmed cell death ligand-1 was associated with better clinical outcome through decreasing tumor-infiltrating lymphocytes in patients with UVM (Zoroquiain et al. 2018). However, there were still very few PCD-related studies in UVM. Therefore, it is necessary to further explore the roles of PCD genes in the progression of UVM.

In our study, the expression patterns, prognostic values and the effects of tumor immune microenvironment on PCD genes were investigated in UVM. The consensus clustering algorithm was utilized to classify UVM patients into two clusters such as cluster1 and cluster2. As the Kaplan–Meier survival curves crossed, our results obtained a shorter OS in UVM patients in cluster2 compared with cluster1, suggesting that the clinical benefit patients were identified by consensus clustering algorithm in cluster1. This seems to be consistent with previous studies (Bao et al. 2022; Shao et al. 2021). Bao et. al showed a longer OS of lower-grade gliomas patients in cuproptosis-related genes cluster B using consensus clustering analysis (Bao et al. 2022). The consensus clustering was applied to recognize the hepatocellular carcinoma subtypes and screen the prognostic values of signature (Fu and Song. 2021). Similarly, the consensus clusters in module A were remarkably related to immune-related pathways and showed better OS in UVM (Jin et al. 2021).

Furthermore, we also constructed an eight-gene prognostic signature consisting of CD38, TYROBP, CDH1,BCL3, HMOX1, CTSC, CD5 and CCR5 from PCD genes. We calculated risk score and constructed prognostic PCD related-signature model for prediction of overall survival in UVM patients. The high-risk scores were significantly related to poor overall survival as independent prognostic factors. The recent study showed that the risk scores of cuproptosis associated-signature were negatively correlated with the overall survival in UVM, revealing its independent prognostic value (Huang et al. 2023). Ferroptosis-mediated modification patterns were also considered as suitable potential biomarkers for UVM prognosis (Jin et al. 2021). Study identified autophagy-related risk signature as independent prognostic ability in UVM (Zheng et al. 2021). The risk signature of sphingolipid metabolism genes was considered to be strongly associated with the prognosis of UVM and showed excellent predictive performance (Chi et al. 2022). Another prognostic six-gene signature was related to glycolysis and immune response as a potential therapeutic target for UM patients (Liu et al. 2021). The similar outcomes were found in multiple other cancers, including breast cancer, lung cancer and colon cancer (Cardoso et al. 2016; Wu et al. 2021; Zhang et al. 2019).

Among these risk markers, CD38 is a non-lineage-restricted, type II transmembrane glycoprotein and belongs to the ADP-ribosyl cyclase family (Tohgo et al. 1994). The molecular function for CD38 gene is hydrolase activity and NAD + nucleosidase activity (Moreschi et al. 2006; Takasawa et al. 1993). Studies have been showed that CD38 inhibited the metabolism and proliferation in prostate cancer (Chmielewski et al. 2018). TYROBP encodes a transmembrane signaling polypeptide which contains an immunoreceptor tyrosine-based activation motif (Mulrooney et al. 2013). TYROBP were negatively associated with prognosis of GC patients (Jiang et al. 2020). TYROBP regulated macrophage activation in osteosarcoma (Liang et al. 2021). TYROBP expression predicted poor prognosis and high tumor immune infiltration in patients with low-grade glioma (Lu et al. 2021). CDH1 is a classical cadherin of the cadherin superfamily (Meigs et al. 2002). CDH1 overexpression inversely correlated with immune infiltration in bladder cancer (Fan et al. 2022). Moreover, CDH1 mutations were identified as independent predictors of poor progression-free survival and primary resistance to immunotherapy in gastrointestinal cancer (Wang et al. 2022b). In UVM, proteomic analysis showed that CDH1 was dysregulated between metastasizing and non-metastasizing patients (Jang et al. 2021). Another study had screened CDH1 as predictive of primary uveal melanoma metastasis (Demirci et al. 2013). BCL3 is a proto-oncogene candidate and forms a part of the autoregulatory loop (McKeithan et al. 1994). Study have showed that BCL3 was high expression in esophageal squamous cell carcinoma (Soares-Lima et al. 2021) and exerted an oncogenic function by regulating STAT3 in human cervical cancer (Zhao et al. 2016). HMOX1 is an essential enzyme in heme catabolism and releases the central heme iron chelate as ferrous iron (Lightning et al. 2001). Upregulation of HMOX1 promoted Fe^2+^ accumulation and overcame cisplatin resistance in ovarian cancer (Ni et al. 2023). Similarly, The HMOX1 was used to construct 8-PCD signature risk model and our results revealed that higher risk scores may be associated with more sensitive to cisplatin (r = 0.7571, *P* = 1.132e-15). However, the molecular mechanism of HMOX1 in progression of UVM cells remained obscure. To investigate the influence of HMOX1 on proliferation and cell apoptosis in UVM cells, the CCK-8 and western blot were performed by using human UVM cells. Our results demonstrated that overexpression of HMOX1 inhibited proliferation and promoted apoptosis of UVM cells, which was identified the consistent similarities in the roles of the chronic lymphocytic leukemia (Amigo-Jimenez et al. 2016). Furthermore, HMOX1 silencing promoted the proliferation and weakened the apoptosis via regulating BAX and BCL-2 expression in UVM cells, which was found in literature (Ren et al. 2022). CTSC encodes a member of the peptidase C1 family and lysosomal cysteine proteinase (Korkmaz et al. 2019). The expression of CTSC in glioma was higher than that in no-cancerous cells (Cheng et al. 2022). In this study, the results of immune infiltration analysis identified CTSC was positively correlated with Tgd cells (r = 0.77, *P* = 2.5e-16), Th2 cells (r = 0.74, *P* = 3.5e-14), CD8 Tcm (r = 0.66, *P* = 8.7e-11) and CD8 Tem (r = 0.57, *P* = 5.4e-08) (Supplementary Table 4). While the result of scRNA-seq revealed that CTSC was more accumulated in the Th1 cells (Supplementary Fig. 3B). Yu et al. showed that CTSC led to accumulation of the tumour infiltrated CD8 + T cells (Yu et al. 2023). CD5 is a member of the scavenger receptor cysteine-rich superfamily and may act as a receptor to regulate T-cell proliferation (Lee et al. 2010). CD5 mediated tumor survival through JAK-STAT signaling pathways in diffuse large B-cell lymphoma (Yang et al. 2023). CCR5 is a member of the beta chemokine receptor family and expressed by T cells and macrophages (Sharapova et al. 2018). CCR5 has been reported to serve as a potential diagnostic marker and therapeutic target for tumor budding in colorectal cancer (Gao et al. 2022b). Although there is a lack of research on CD5 and CCR5 in the progression of UVM, numerous evidences suggested that immune microenviroment played vital roles in immunoregulation of UVM (Garcia-Mulero et al. 2021; Jin et al. 2021; Wang et al. 2020). Patients with better survival showed higher levels of naïve B cells, resting T cells CD4 memory and lower levels of CD8 + T cells and gamma delta T cells (Jin et al. 2021). It was consistent with our results that accumulation of CD8 Tcm (*P* = 5.67e-05), CD8 Tem (*P* = 0.0006188) and Tgd cells (*P* = 9.2e-06) were found in high-risk group with poor prognosis. The scRNA-seq data showed that the dendritic cells and cytotoxic T cells were related to the prognosis of UVM (Gao et al. 2022a). Similarly, our results revealed that the high levels of DC showed poor prognosis (*P* = 0.0054625). In our immune infiltration results, HMOX1 was the most positively correlated with macrophages (r = 0.753, *P* = 3.7e-15) and followed by aDC (r = 0.748, *P* = 7.3e-15, Supplementary Table 4). Interestingly, the results of scRNA-seq demonstrated high HMOX1 expression positive rate on macrophages and Th1 cells, suggesting HMOX1 might involve in the progression of UVM through macrophages activation. Tang et al. showed that there was significant positive association between macrophage and SPTBN1 expression in UVM (Tang et al. 2023). Immune infiltration could also screen previously unrecognized diversity of cell types through scRNA-seq, such as checkpoint marker LAG3, rather than programmed cell death ligand-1 or CTLA4 (Durante et al. 2020). In future, larger scale scRNA-seq in clinical UVM samples and flow cytometry experiments should be implemented to verify our results.

To explore whether PCD signature scores could predict the therapeutic efficacy of chemotherapy in UVM, GSDC database were used to analyze the correlation between risk scores and chemotherapeutic drugs. Our results showed the most significant positive correlation between risk scores and cisplatin, suggesting higher risk scores was associated with more sensitive to cisplatin. The similar results were found in potential therapeutic implications in high-risk in patients with pancreatic ductal adenocarcinoma using GDSC database (Liu et al. 2023). Furthermore, the sensitivity of UVM to cisplatin was enhanced by inhibiting autophagy-related gene expression (He et al. 2023). Calcium electroporation with Bleomycin induced a significant increase of the apoptosis and a reduction of vascularization in UVM xenograft model (Tsimpaki et al. 2024). To predict clinical response to immune checkpoint blockade based on pre-treatment tumor profiles, the TIDE method was used to predict the response of high- and low-risk score UVM patients to immune checkpoint blockade therapy. Our results showed that TIDE score was significantly lower in high-risk group, suggesting the better immunotherapy responsiveness in the high-risk group. These results were consistent with those of related study, and suggested that TIDE scores were higher in the low-risk UVM group (Nan et al. 2023). On the contrary, bladder cancer patients in low-risk group were more sensitive to immunotherapy (Wu et al. 2022). Furthermore, apoptotic cell death contributed to inducing immune responses in uveal melanoma cells (Carlring et al. 2003).

Therefore, there were serve limitation in our study. Firstly, the public databases were inevitably limited by an inherent case selection bias. More large-scale prospective were needed to confirm our results. Furthermore, although the roles of HMOX1 in proliferation and apoptosis were investigated in UVM cells, more functional experiments in vivo or in vitro are further needed to verify the roles of PCD-related signature genes.

## Conclusion

We established a novel risk model for UVM patients, constituting by an 8-PCD signature (CCR5, HMOX1, CTSC, CD5, BCL3, CDH1, TYROBP and CD38). We also found that the PCD prognostic signature was related to tumor immune microenvironment, clinical features, drug sensitivity, and prognosis. The effects of HMOX1 on the proliferation and apoptosis were investigated in UVM cells. Moveover, fer-1 completely protected UVM cells from apoptosis and necrosis caused by OE-HMOX1. Our results provided insight into the potential clinical implications of PCD prognostic signature, inferring that PCD signature genes may be the potential therapeutic target for UVM patients.

### Supplementary Information

Below is the link to the electronic supplementary material.

Supplementary file1 (TIF 1503 KB)

Supplementary file2 (TIF 3993 KB)

Supplementary file3 (TIF 1396 KB)

Supplementary file4 (XLSX 41 KB)

Supplementary file5 (XLSX 10 KB)

Supplementary file6 (XLSX 9 KB)

Supplementary file7 (XLSX 14 KB)

## Data Availability

The datasets used and analyzed during the current study are available from the corresponding author upon reasonable request.
