# Supplementary figures and images for: Programmed Cell Death-Related Gene Signature Associated with Prognosis and Immune Infiltration and the Roles of HMOX1 in the Proliferation and Apoptosis were Investigated in Uveal Melanoma
Source: Genes Genomics. 2024 May 20;46(7):785–801. doi: 10.1007/s13258-024-01521-x (PMC11208274; doi:10.1007/s13258-024-01521-x)

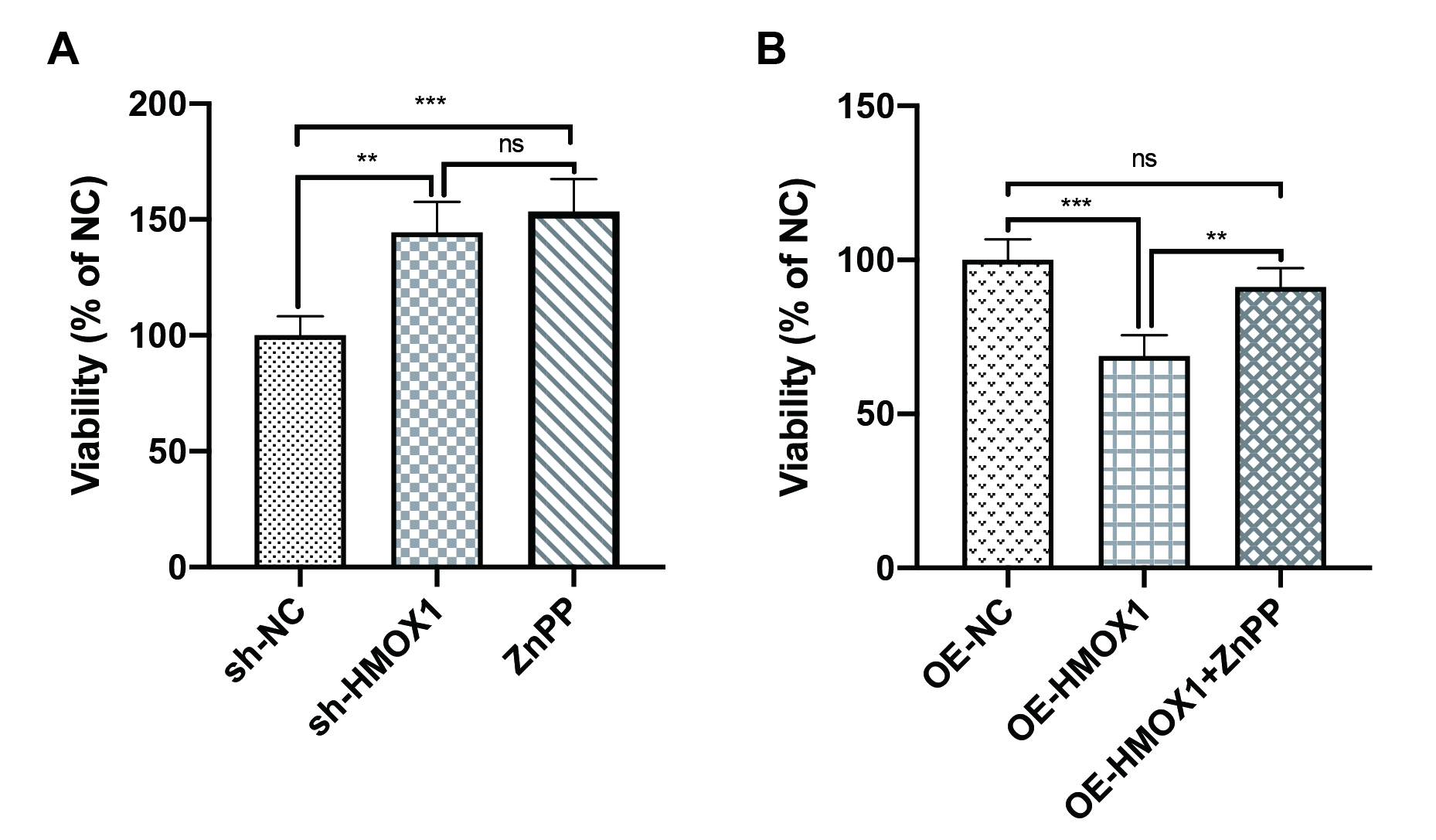

Supplement: Supplementary file 1 — Supplementary file1 (TIF 1503 KB) [file 13258_2024_1521_MOESM1_ESM.tif]

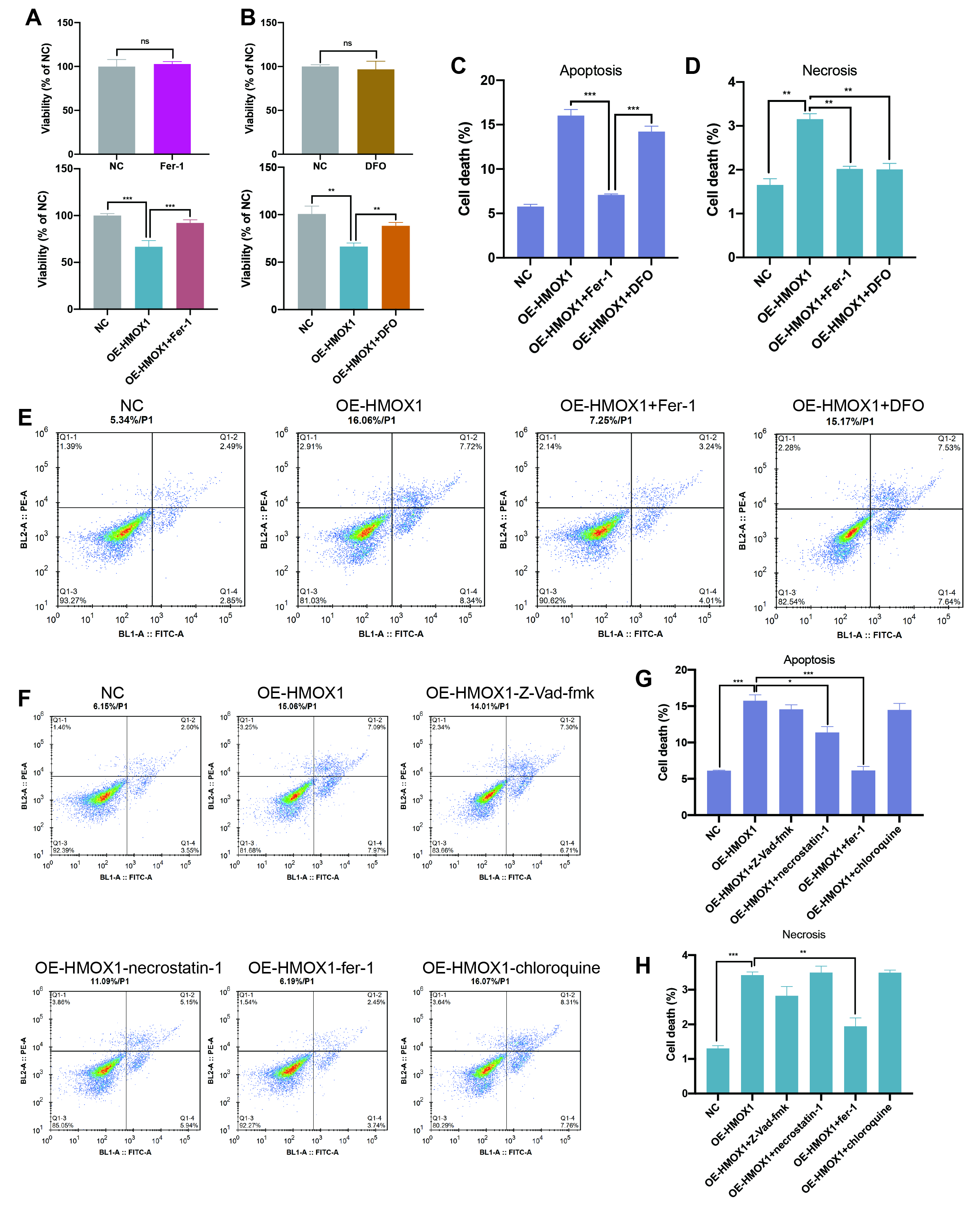

Supplement: Supplementary file 2 — Supplementary file2 (TIF 3993 KB) [file 13258_2024_1521_MOESM2_ESM.tif]

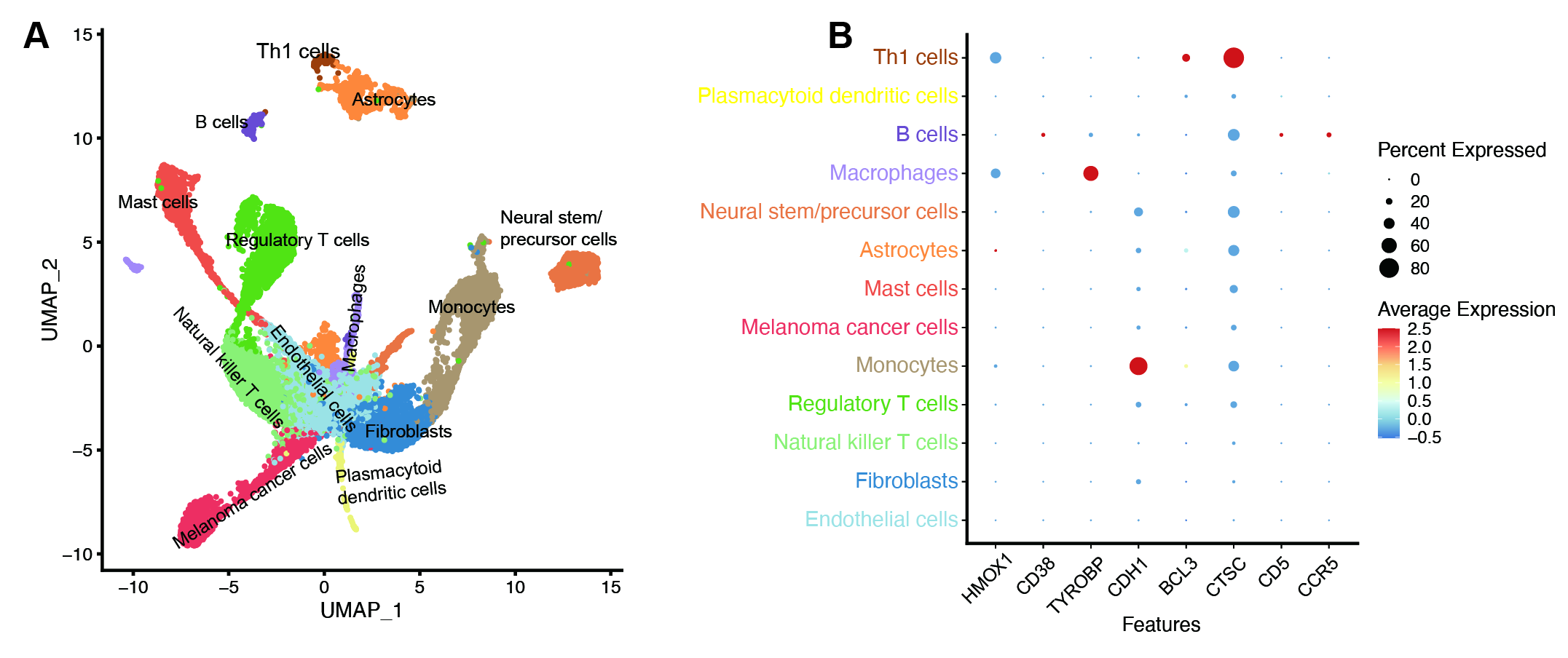

Supplement: Supplementary file 3 — Supplementary file3 (TIF 1396 KB) [file 13258_2024_1521_MOESM3_ESM.tif]
